# The multiple sclerosis rating scale, revised (MSRS-R): Development, refinement, and psychometric validation using an online community

**DOI:** 10.1186/1477-7525-10-70

**Published:** 2012-06-18

**Authors:** Paul Wicks, Timothy E Vaughan, Michael P Massagli

**Affiliations:** 1 PatientsLikeMe Inc, 155 Second Street, Cambridge, MA 02141, USA

**Keywords:** Multiple sclerosis, Patient-reported outcomes, Disability, MS relapse, Online research, Internet research

## Abstract

**Background:**

In developing the PatientsLikeMe online platform for patients with Multiple Sclerosis (MS), we required a patient-reported assessment of functional status that was easy to complete and identified disability in domains other than walking. Existing measures of functional status were inadequate, clinician-reported, focused on walking, and burdensome to complete. In response, we developed the Multiple Sclerosis Rating Scale (MSRS).

**Methods:**

We adapted a clinician-rated measure, the Guy’s Neurological Disability Scale, to a self-report scale and deployed it to an online community. As part of our validation process we reviewed discussions between patients, conducted patient cognitive debriefing, and made minor improvements to form a revised scale (MSRS-R) before deploying a cross-sectional survey to patients with relapsing-remitting MS (RRMS) on the PatientsLikeMe platform. The survey included MSRS-R and comparator measures: MSIS-29, PDDS, NARCOMS Performance Scales, PRIMUS, and MSWS-12.

**Results:**

In total, 816 RRMS patients responded (19% response rate). The MSRS-R exhibited high internal consistency (Cronbach’s alpha = .86). The MSRS-R walking item was highly correlated with alternative walking measures (PDDS, ρ = .84; MSWS-12, ρ = .83; NARCOMS mobility question, ρ = .86). MSRS-R correlated well with comparison instruments and differentiated between known groups by PDDS disease stage and relapse burden in the past two years. Factor analysis suggested a single factor accounting for 51.5% of variance.

**Conclusions:**

The MSRS-R is a concise measure of MS-related functional disability, and may have advantages for disease measurement over longer and more burdensome instruments that are restricted to a smaller number of domains or measure quality of life. Studies are underway describing the use of the instrument in contexts outside our online platform such as clinical practice or trials. The MSRS-R is released for use under creative commons license.

## Background

Multiple Sclerosis (MS) is a neurological condition characterised by lesions of myelin sheaths encapsulating the neurons of the brain, spine, and optic nerve, causing transient or progressive symptoms and disability. Measuring MS is challenging; objective measurement requires complex tools (e.g. MRI using an expensive and immobile device), experience (e.g. examination from a specialist neurologist), and/or significant time to complete (e.g. MS Functional Composite, 15 minutes of testing requiring special equipment [1]). Patient-perceived symptoms can fluctuate seasonally [2], daily, hourly, or even in response to variations in temperature [3]; they may be unmasked only on specific tasks, and they may involve complex systems such as vision, cognition, sexual function, and bladder function.

The PatientsLikeMe online data platform (http://www.patientslikeme.com) was built to allow patients with life-changing illnesses to share data about their experiences of symptoms and disability through structured data collection [4]. Use of the system has shown benefit through improved health literacy, better communication with healthcare professionals, and development of a peer support network [5,6]. The platform has proved useful in developing other patient-reported outcomes (PROs) using patients' own language [7,8].

In expanding the platform in 2007 to include MS, a number of instruments were considered. The MS Impact Scale (MSIS-29, [9]) was not intended solely to measure MS disability; it also included health-related functional impact (e.g. limitations in social and leisure activities). The MS Walking Scale (MSWS-12) has the obvious limitation of focusing only on walking [10,11]. The North American Research Committee on Multiple Sclerosis (NARCOMS) patient registry has developed validated performance scales (PS) [12,13] in areas including walking, fatigue, cognition, and vision. The PS have clearly defined anchor points for each response; but the instrument is long (about 2,500 words), and the inconsistent response format requires close reading to avoid confusion and erroneous reporting — a potential challenge for patients with cognitive issues and fatigue. NARCOMS has also used the patient-determined disease steps (PDDS) [14], which resembles a patient-reported form of the Expanded Disability Status Scale (EDSS) and may have some of the same limitations as that instrument [15]. A newly developed measure of activity limitation, symptoms, and quality of life, the Patient-Reported outcome Indices for Multiple Sclerosis (PRIMUS [16]), has been published and is starting to be used in trials [17].

In the absence of an agreed-upon “gold standard” PRO, we collaborated with an MS specialist to develop the MS Rating Scale (MSRS). This paper describes the development of the original MSRS, as well as work to revise the scale through cognitive debriefing to produce a revised version (MRSR-R), data on psychometric performance, and comparisons with other patient-reported MS scales.

## Methods

### MS Rating Scale (MSRS) Development

**Figure 1:**
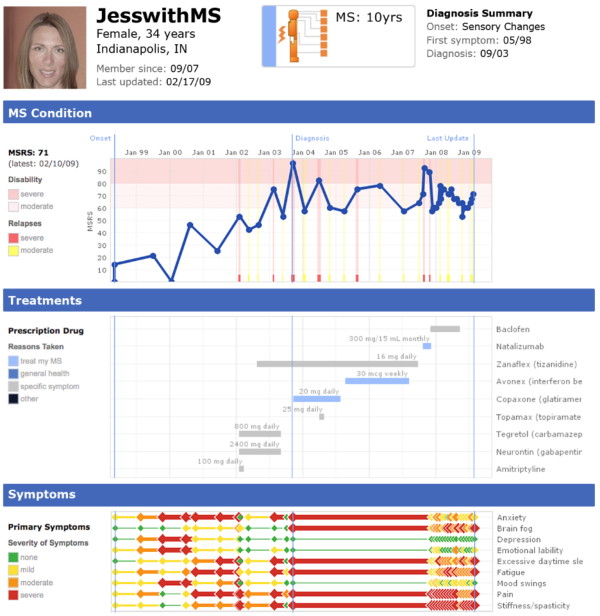
**Profile of a sample MS patient featuring rich data and history.** Written informed consent was obtained from the patient for publication of this report and any accompanying images.

The objective of the MSRS was to accurately quantify the level of MS-relevant disability experienced by patients across a range of domains affected by demyelinating lesions. Observation of an MS specialist's clinic at King's College Hospital in London identified seven domains routinely asked about in clinical practice as part of a "top to toe" clinical interview. These domains were intended to reflect the degree of lesion burden for nervous system regions enervating the region of interest (e.g. optic nerves for “vision”). We adapted a modified scoring scheme from the Guy’s Neurological Disability Scale (GNDS [18]), which took a relatively consistent approach to scoring each domain, using the first four levels of disability (“0 - Normal status”, “1 - Symptoms causing no disability”, “2 - Mild disability not requiring help from others”, “3 - Moderate disability requiring help from others”) and the final level (4 - “Total loss of function, maximal help required”). A consistent scoring scheme was preferred in order to minimize response burden and encourage repeated entry of data longitudinally. Total score was the sum of the 7 items, with a range of 0–28. For website display the patient profile rescales the score to a 0–100 scale (Figure 1). Remaining domains from the GNDS, such as “mood” (split into anxiety and depression), bladder, bowel, sexual dysfunction, fatigue and spasms, were integrated into the existing PatientsLikeMe symptoms system and rated by patients as “none”(0), “mild” (1), “moderate” (2), or “severe” (3).

The resulting scale, the MSRS, was felt to have the advantage of tapping a range of important domains for MS patients, not solely focused on walking but including other aspects of function that might be important to monitor over time. Using the MSRS, patients are easily able to create a longitudinal record of their experience of MS to share with others and to help understand the impact of their treatments (Figure 1). At the time of survey invitation (Fall 2010), 15,219 users had completed at least one MSRS survey, for a total of 72,975 reports on the PatientsLikeMe system. Members join the site understanding that their de-identified data will be used for research as part of the terms of service.

### Review and Revision of the MSRS

#### Cognitive Interviews

To test that the instrument captured all domains considered relevant by patients and to identify areas for improvement, patients were recruited for interview by private message on the PatientsLikeMe platform. All patients were local to Boston, Massachusetts and were selected to represent diverse clinical experience. Patients were offered a $50 honorarium for their participation, which took approximately 2 hours. Feedback served as the basis for a revised MSRS (MSRS revised, MSRS-R).

The wording of MSRS response options was clarified, defining “disability” more clearly and changing the highest response category from “total” to “severe” disability, along with minor text changes. (see underlined text in Table 1 for revisions). The domain “upper limb function” was clarified to ”using your arms and hands”, and “bowel or bladder” dysfunction was added as a domain of functional impairment (see Table 2 for revisions).

**Table 1:** Original and revised MSRS anchor points

**Original MS Rating Scale (MSRS)**	**MSRS, Revised (MSRS-R)**
**No Symptoms -** No symptoms or disability in this specific area	**No Symptoms -** No symptoms or disability in this specific area
**None -** Aware of symptoms but no functional disability	** Some Symptoms, No Disability -** Aware of symptoms but no limits on my activities
**Mild -** Mild disability but not requiring help from others	** Mild Disability -** Mild limits on my activities, but I do not need help from others or use other aids
**Moderate -** Moderate disability that requires some help from others	** Moderate Disability -** Moderate limits on my activities, and I sometimes need help from others or use other aids
**Total Disability -** Total disability and help always required	** Severe Disability -** Severe limits on my activities, and I usually need help from others or use other aids

**Table 2:** Original and revised MSRS items

**Original MS Rating Scale (MSRS)**	**MSRS, Revised (MSRS-R)**
Walking	Walking
Upper Limb Function	Using your arms and hands
Vision	Vision (with glasses or contacts if you use them)
Speech	Speaking clearly
Swallowing	Swallowing
Thinking/Memory/Cognition	Thinking, Memory, or Cognition
Sensation/Burning/Pain	Numbness, Tingling, Burning Sensation or Pain
n/a	Bowel or bladder

#### Online survey

The PatientsLikeMe survey system was used to test the psychometric properties of the MSRS-R (incorporating feedback from the cognitive debriefings). The survey consisted of the MSRS-R, a fixed list of MS symptoms, and a report about the patient's most recent relapse. If they chose to report on that relapse, they were asked for start- and end-dates of the relapse, to rate the severity of the relapse (“mild”, “moderate”, or “severe”), and whether the relapse had required hospitalization or treatment with steroids, or resulted in any permanent loss of function. The patient then used the MSRS-R to describe their level of disability when they were feeling *worst* during the relapse.

The remaining sections of the survey were composed of scales identified as being used as secondary outcome measures in clinical trials: the MSIS-29, NARCOMS PS, PDDS, PRIMUS, and MSWS-12. Quality-of-life instruments such as the MSQOL-54 were not used because of their predominant focus on QOL as opposed to disability. [Note: The PRIMUS consists of 3 components – 22 symptoms, 15 activities, and 22 quality-of-life statements. We made an error in implementing the quality-of-life component and included only the first 12 items, but fortunately these items sample the full range of item locations on the quality-of-life scale, as described by the PRIMUS developers [16]]. Those who had completed the survey within one week of initial invitation were asked to complete a 1-week retest, which included only the PatientsLikeMe measures. For this follow-up survey the patients were asked to respond retrospectively about how they were feeling *at the time of the first survey*.

Upon site registration, PatientsLikeMe users agree that they may be asked to participate in research; as a research study using only online questionnaires with minimal risk, IRB approval was not sought for this study. However, in accordance with the Declaration of Helsinki, participants were informed about the aims of the study, were given the option to opt-in without incentive and opt-out without any negative consequences. In order to target active users, we invited patients accessing their accounts during the 90 days prior to 24 August 2010. Five days after the initial invitation, a reminder was sent to all invited patients who had not yet completed the survey. At that point an invitation to the retest was also sent to all who had completed the baseline survey within the first six days of the field period. Retest participants also received a reminder 5 days after the retest invitation.

#### Statistical Analysis

Data were analyzed using Statistical Package for the Social Sciences (SPSS) V20. Descriptive statistics document the distribution of responses and measurement properties of the MSRS-R. Principal component analysis was used to identify the structure of the MSRS-R. The number of factors was left unconstrained, with eigenvalues >1 initially considered worthy of further interest. We also conducted a parallel analysis, using the procedure of Horn [19] and SPSS syntax [20], in order to compare the magnitude of observed eigenvalues against that generated by random arrangements of the same data.

Internal consistency was assessed using item-to-item Spearman correlations and Cronbach’s alpha, which should be above 0.7 to be considered adequate. Concurrent validity was tested using correlations between the MSRS-R and other scales, in addition to subscales of the MSIS-29, MSWS-12, and PRIMUS. Test-retest reliability was assessed first with Spearman correlations and then with a Bland-Altman plot.

Known-group validity was assessed by comparing the MSRS-R scores of patients grouped by level of impairment on the self-reported PDDS, which is known to correlate highly with the EDSS, a widely used clinician-rated scale in MS trials. In addition, we used the patient’s estimate of the number of relapses they had experienced in the past two years, on the basis that relapses in relapsing-remitting MS contribute to a worsening burden of disability [21]. ANOVA (with Bonferroni corrected post-hoc tests) was used to compare MSRS-R scores in the groups, and it was hypothesized that patients in more severe PDDS groups or with a higher number of relapses in the past two years would have worse (higher) MSRS-R scores. Given the prominence of walking measurements in MS, we also performed similar analyses for the MSRS-R walking item. Other between-group differences were assessed using ANOVA, Student’s *t*-test, and Kruskal-Wallis tests as appropriate. Clinician-assessed validity and responsiveness to change are the subjects of future investigations.

## Results

### Responder characteristics

Data reported here describe patients who self-reported a diagnosis of relapsing-remitting MS (RRMS), but data were also collected for patients with other subtypes (not reported here). The main survey was launched on 8 September 2010. 4,382 invitations were issued to RRMS patients; complete responses were received from 816 RRMS patients (18.6%); incomplete responses were received from 156 patients (3.6%, only complete responses are described here), and opt-outs from 121 (2.8%). The remaining 3,289 (75.1%) of patients made no response to the survey invitation.

All prospective members of the site are invited to add their age and sex to their profile, but not all had chosen to do so by the time of survey. Using available profile data from non-completers, patients who completed the baseline survey were around 3 years older than non-completers (see Table 3). The groups differed significantly on sex, although this difference became a non-significant trend after removing patients without ascertained sex (*X*^2^(1) = 3.476, p = .063). Baseline completes were also more likely to have reported more relapses on their profile in the 2 years prior to the survey, but this may represent different levels of engagement on the website rather than true disease severity; a similar explanation may underlie for the similar pattern for most recent MSRS score from the patients’ profiles.

**Table 3:** Description of patient samples including completes and non-completes

	**Baseline (4,382 eligible)**			**1-Week Retest (391 eligible)**		
	**Non-Completes**	**Completes**	**Sig.**	**Non-Completes**	**Completes**	**Sig.**
**N of cases**	3566	816		199	192	
**Sex**						
Female (%)	2715 (76.1)	619 (75.9)	*X*^2^ = 15.25	151 (75.9)	144 (75.0)	*X*^2^ = 2.22
Male (%)	622 (17.4)	170 (20.8)	df = 2	42 (21.1)	46 (24.0)	df = 2
Not ascertained (%)	229 (6.4)	27 (3.3)	p < .001	5 (2.0)	2 (1.0)	p = .329
**Age in years**						
Mean (SD)	42.9 (9.9)	45.9 (9.8)	t = 7.61	45.5 (9.9)	46.5 (9.5)	t = .989
Median (IQR)	43.0 (36–50)	47.0 (39–53)	df = 4,102	45.0 (38–53)	46.0 (40–53)	df = 377
Range	15 - 81	19 - 73	p < 0.001	19 – 73	21 - 67	p = .323
Not ascertained (%)	251 (7.0)	27 (3.3)		9 (4.5)	3 (1.6)	
**Years since MS diagnosis**						
Mean (SD)	6.5 (6.2)	6.6 (6.6)	t = .79	5.7 (7.7)	6.7 (6.3)	t = −.106
Median (IQR)	4.0 (2–9)	4.0 (2–9)	df = 4,326	4.0 (2–9)	5.0 (2–9.8)	df = 388
Range	0 - 39	0 - 52	p = .430	0 - 52	0 - 37	p = .916
Not ascertained (%)	50 (1.4)	4 (0.5)		1 (0.5%)	0 (0.0)	
**Number (%) of reported relapses in previous 2 years**						
0	2440 (68.4)	471 (57.7)	*X*^2^ = 53.82	119 (59.8)	108 (56.2)	*X*^2^ = 6.14
1	719 (20.2)	182 (22.3)	df = 4	45 (22.5)	44 (22.9)	df = 4
2	216 (6.1)	85 (10.4)	p < 0.001	20 (10.1)	15 (7.8)	p = .189
3	106 (3.0)	35 (4.3)		5 (2.5)	15 (7.8)	
4 or more	85 (2.4)	43 (5.3)		10 (5.0)	10 (5.2)	
**PDDS group (%)**						
normal	n/a	189 (23.2)	n/a	46 (23.1)	42 (21.9)	*X*^2^ = 4.55
mild disability	n/a	107 (13.1)		26 (13.1)	26 (13.5)	df = 4
moderate or gait disability	n/a	242 (29.7)		60 (30.2)	46 (24.0)	p = .225
early cane	n/a	143 (17.5)		35 (17.6)	32 (16.7)	
late cane, bilateral support, wheelchair, or bedridden	n/a	135 (16.5)		32 (16.1)	46 (24.0)	
**Most recently entered profile MSRS (%)**						
not ascertained	314 (8.8)	25 (3.1)	*X*^2^ = 47.515	11 (5.5)	1 (0.5)	*X*^2^ = 10.24
0 to 9	341 (9.6)	84 (10.3)	df = 7	24 (12.1)	25 (13.0)	df = 7
10 to 19	511 (14.3)	126 (15.4)	p < 0.001	23 (11.6)	31 (16.1)	p = .175
20 to 29	544 (15.3)	131 (16.1)		28 (14.1)	28 (14.6)	
30 to 39	677 (19.0)	132 (16.2)		33 (16.6)	33 (17.2)	
40 to 49	413 (11.6)	87 (10.7)		20 (10.1)	16 (8.3)	
50 to 59	417 (11.7)	134 (16.4)		33 (16.6)	35 (18.2)	
60 or higher	97 (11.9)	97 (11.9)		27 (13.6)	23 (12.0)	

A little over half of patients reported a recent relapse (52%, N = 424/816). Duration since relapse was distributed between 33% (N = 140/424) reporting one within the month prior to survey completion, 23% (N = 97/424) reported a relapse between one and three months prior, and 44% (N = 187/424) reporting a relapse three or more months ago. The vast majority of relapses (98.7%) were reported from the past decade (2000–2010).

On 14 September, invitations for the 1-week retest were sent to the 391 patients who had completed the survey by this point. 192 RRMS patients (49.1%) completed the retest survey; 27 (6.9%) provided incomplete answers, and 10 (2.6%) opted out at this stage. The remaining 162 (41.4%) made no response to the retest survey invitation. Participants who completed the retest did not differ on sex, age, disease duration, or disease severity from other eligible participants who took the baseline survey (Table 3), or from those who had not completed the baseline survey in time to be eligible for the retest (not shown). Both surveys were closed to further participation on 23 September 2010.

### MSRS-R Psychometric Characteristics

The revised MSRS-R measure added “bowel or bladder” as a functional area. The revised measure also characterized levels of impairment using more patient-friendly terms around “activity limitation” in contrast to “functional disability”, and characterized the most disabled state as “severe” rather than “total disability”. Although we did not design the study to compare severity of disability using the original MSRS and the MSRS-R, we did have a small number of respondents (n = 211) who had used the original MSRS to populate their site profile within a month of completing the baseline MSRS-R for this study. Table 4 shows somewhat greater use of the extreme disability category (“severe”) for the MSRS-R compared with the extreme disability category (“total disability”) for the original MSRS.

**Table 4:** Proportion of patients in most extreme disability category in original (MSRS) and revised (MSRS-R) scales

**MSRS Item**	**MSRS: % of patients reporting “total disability”**	**MSRS-R: % of patients reporting “severe disability”**
Walking	8	18
Upper limb / Arms and Hands	1	1
Vision	1	3
Speaking	0	1
Swallowing	0	1
Thinking, memory, or cognition	2	4
Numbness, tingling, burning sensation, pain	8	13

### Factor analysis

We assessed the dataset for suitability for factor analysis. Given the baseline respondent sample size of 816, we had approximately 103 participants per variable. Correlations between items were all above ρ = 0.3. Bartlett’s test of sphericity [22] was significant at p < 0.001, supporting the factorability of the correlation matrix. The Keyser-Meyer-Olkin value of sampling adequacy was 0.884, exceeding the recommended value of 0.6 [23,24]. PCA revealed a single factor with an eigenvalue of 4.2 accounting for 51.5% of variance; the second highest eigenvalue was 0.9. Further, the results of a parallel analysis [19] using the same dataset showed no components exceeding the corresponding criterion values (8 variables x 816 respondents x 100 replications); the highest eigenvalue produced by the parallel analysis was 2.0. Table 5 shows the MSRS-R item factor loadings.

**Table 5:** Principal component analysis item loadings for a single factor (eigenvalue 4.2, 51.5% of variance)

**MSRS-R Item**	**Factor Loading**
Walking	.639
Using your arms and hands	.770
Vision (with glasses or contacts if you use them)	.708
Speaking clearly	.751
Swallowing	.737
Bowel or bladder	.662
Thinking, memory, or cognition	.739
Numbness, tingling, burning sensation, or pain	.723

### Scale characteristics

Mean MSRS-R score was 10.9 (SD: 6.1) with a median of 10.0 (IQR: 6–15). Only 0.7% of patients scored at floor (MSRS = 0), and no patients scored at ceiling (0%, MSRS = 32.) Table 6 shows the distribution of item scores; walking (66.1%) and sensory aspects (60.2%) were the domains most frequently associated with some level of disability; speech (21.5%) and swallowing (16.6%) were the least frequently affected.

**Table 6:** MSRS-R item distributions

**Current MSRS-R (N = 816)**	**No Symptoms**	**Some symptoms, No Disability**	**Mild Disability**	**Moderate Disability**	**Severe Disability**
	**No symptoms or disability in this specific area**	**Aware of symptoms but no limits on my activities**	**Mild limits on my activities, but I do not need help from others or use other aids**	**Moderate limits on my activities and I sometimes need help from others or use other aids**	**Severe limits on my activities and I usually need help from others or use other aids**
**Walking**	12.4%	21.6%	24.4%	33.7%	8.0%
**Using your arms and hands**	26.8%	33.5%	25.4%	13.5%	0.9%
**Vision (with glasses or contacts if you use them)**	35.0%	31.0%	19.2%	12.7%	2.0%
**Speaking Clearly**	47.2%	31.3%	15.8%	5.6%	0.1%
**Swallowing**	55.6%	27.7%	12.6%	3.9%	0.1%
**Bowel or bladder**	25.5%	27.7%	26.3%	16.4%	4.0%
**Thinking, Memory, or Cognition**	16.7%	32.5%	26.7%	19.9%	4.3%
**Numbness, Tingling, Burning Sensation or Pain**	11.0%	28.7%	27.2%	24.1%	8.9%

### Internal consistency

Cronbach’s alpha (.86) indicated acceptable internal consistency. Item convergent validity as assessed by correlations between items and total score was also acceptable, ranging from Spearman’s rho of ρ = 0.68 for walking to ρ = 0.77 for using arms and hands.

### Test-retest

**Figure 2:**
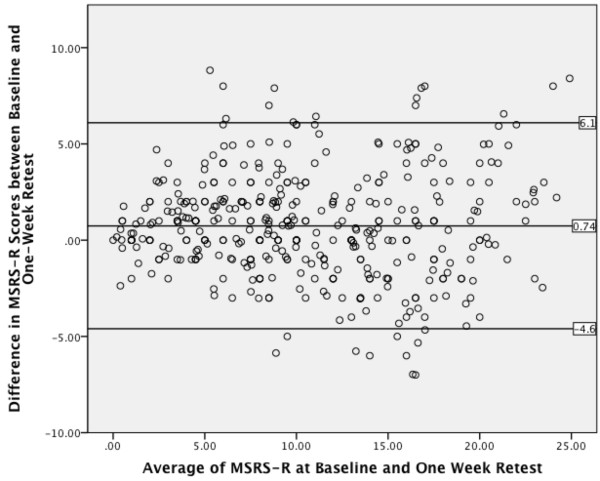
Bland-Altman plot for baseline and 1-week retest MSRS-R.

A Bland-Altman plot of the differences in total score at baseline and 1-week retest (N = 192) showed no systematic pattern and no outliers (see Figure 2). The plot showed 182 of 192 cases (95%) lie within two standard deviations of the mean (mean difference = 0.74, SD: 2.7, Limits of agreement (+/− 2SD): -4.6 – 6.1). Examination of item-level differences using a Wilcoxon signed rank test revealed significant differences only for the “Numbness, Tingling, Burning Sensation, or Pain” item (z = −4.438, p < 0.001) with a small effect size (ρ = .23) and mean difference of 1.6 points (SD: 1.1).

### Concurrent validity

The MSRS-R total score was correlated significantly (p < 0.001) with all comparable measures using Spearman’s rho: PRIMUS symptoms subscale (ρ = .75), MSIS-29 physical subscale (ρ = .74), PRIMUS activities subscale (ρ = .69), MSWS-12 (ρ = .66), PDDS (ρ = .62), MSIS-29 psychological subscale (ρ = .60), and PRIMUS QOL subscale (ρ = .59). MSRS-R total scores with the NARCOMS PS varied from ρ = .44 for depression to ρ = .63 for cognitive; but as the NARCOMS PS do not produce a total score, an item-by-item analysis was performed. Table 7 shows the correlations between MSRS-R domains and NARCOMS PDDS and PS item scores; correlations were highest for walking (ρ = .84), with the remainder of correlations between MSRS-R domains and their relevant PS item scores around ρ = .7, indicating good agreement.

**Table 7:** Correlations (Spearman’s rho) between MSRS-R domains and PDDS and NARCOMS Performance Scales (p < 0.001)

	**NARCOMS**											
**MSRS-R Domains**	**PDDS**	**Mobility**	**Hand**	**Vision**	**Fatigue**	**Cognitive**	**Bladder/Bowel**	**Sensory**	**Spasticity**	**Pain**	**Depression**	**Tremor**
Walking	0.84	0.84	0.43	0.25	0.44	0.27	0.44	0.41	0.55	0.40	0.25	0.53
Using your arms and hands	0.48	0.47	0.70	0.38	0.48	0.44	0.28	0.56	0.50	0.50	0.32	0.49
Vision (with glasses or contacts if you use them)	0.28	0.28	0.35	0.71	0.35	0.42	0.26	0.36	0.32	0.40	0.32	0.32
Speaking Clearly	0.28	0.27	0.42	0.38	0.37	0.47	0.23	0.32	0.28	0.36	0.27	0.35
Swallowing	0.32	0.32	0.39	0.36	0.39	0.43	0.30	0.38	0.33	0.39	0.30	0.35
Bowel or bladder	0.48	0.46	0.30	0.30	0.37	0.34	0.71	0.35	0.36	0.32	0.25	0.36
Thinking, Memory, or Cognition	0.28	0.26	0.37	0.41	0.49	0.77	0.28	0.38	0.34	0.44	0.42	0.42
Numbness, Tingling, Burning Sensation or Pain	0.45	0.42	0.48	0.36	0.51	0.45	0.31	0.72	0.53	0.67	0.38	0.47

### Known group validity

We hypothesized that higher total MSRS-R scores (worse disability) would be observed for those with worsening PDDS status and a higher number of relapses.

Between-groups differences on MSRS-R by PDDS levels were found to be significant using ANOVA (F(7,808) = 77.250, p < .001). Post-hoc Bonferroni tests of all pair-wise comparisons revealed significant differences between MSRS-R for normal and mild PDDS from all other PDDS levels (p < .001). “Moderate” and “Gait disability” PDDS levels were not significantly different from one another (p > 0.05). Higher levels of mobility impairment (“early cane”, “late cane”, “bilateral support”) differed significantly from “normal”, “mild”, and “moderate” disability, but not from each other (p > 0.05). “Use of a wheelchair or scooter” differed only from “normal” or “mild” disability (p < 0.05) on the MSRS-R. No respondents endorsed the most severe category on the PDDS (“Bedridden”). Examination of individual MSRS-R items revealed a much stronger step-wise relationship between PDDS and the MSRS-R walking item than other items, although “bladder & bowel” shows a similar, though less marked pattern (Table 8).

**Table 8:** Comparison of MSRS-R and MSRS-R items by known PDDS groups

**Mean (S.D)**	**n of cases**	**Total MSRS-R (0–32)**	**Walking (0–4)**	**Arms & Hands (0–4)**	**Vision (0–4)**	**Speaking (0–4)**	**Swallowing (0–4)**	**Bowel & Bladder (0–4)**	**Thinking, Memory, Cognition (0–4)**	**Numbness, Tingling, Burning Sensation or Pain (0–4)**
PDDS group										
0 - normal	189	4.9	0.7	0.5	0.6	0.3	0.2	0.7	1.0	1.1
		(3.5)	(0.7)	(0.7)	(0.8)	(0.5)	(0.5)	(0.8)	(0.8)	(0.8)
1 - mild disability	107	8.8	1.4	0.9	0.9	0.7	0.5	1.2	1.5	1.6
		(4.5)	(7.5)	(0.8)	(0.9)	(0.8)	(0.8)	(1.0)	(1.0)	(1.0)
2 - moderate	99	11.8	1.8	1.4	1.5	1.0	0.6	1.4	2.0	2.0
		(4.2)	(0.8)	(0.8)	(1.1)	(0.9)	(0.8)	(1.0)	(1.0)	(1.0)
3 - gait disability	143	12.3	2.4	1.6	1.3	0.9	0.8	1.5	1.8	2.1
		(4.8)	(0.6)	(1.0)	(1.0)	(0.9)	(0.8)	(1.0)	(1.1)	(1.0)
4 - early cane	143	14.1	2.9	1.6	1.4	1.0	0.9	2.0	1.9	2.4
		(5.7)	(0.5)	(1.0)	(1.1)	(1.0)	(0.9)	(1.1)	(1.1)	(1.1)
5 - late cane	90	15.9	3.3	2.0	1.7	1.2	1.1	2.0	2.0	2.6
		(5.3)	(0.5)	(0.9)	(1.3)	(1.0)	(1.0)	(1.0)	(1.2)	(1.1)
6 - bilateral support	31	15.5	3.5	1.9	1.4	1.0	0.9	2.7	1.8	2.4
		(5.4)	(0.5)	(1.1)	(1.1)	(1.0)	(1.1)	(1.0)	(1.0)	(1.2)
7- wheelchair or scooter	14	13.4	3.9	1.4	1.0	0.9	0.9	2.3	1.2	2.1
		(5.5)	(0.4)	(1.3)	(0.3)	(1.1)	(1.0)	(1.2)	(1.2)	(1.7)
8 - bedridden	0	n/a	n/a	n/a	n/a	n/a	n/a	n/a	n/a	n/a

Our second known group comparison consisted of comparing MSRS-R scores against the number of self-reported MS relapses on patients’ profiles for the two years preceding the survey (Table 9). One-way ANOVA showed significant differences for total MSRS-R score (F(4,811) = 6.422, p < 0.001). Two post-hoc Bonferroni tests were significant: between “no relapses reported” and “4 or more” (mean difference: 4.3, 95% CI: 1.6-6.9, p < 0.001) and between “1 relapse” and “4 or more” (mean difference: 3.3, 95% CI: 0.5-6.1, p < 0.001). We also checked whether PDDS were significantly different across recent burden of relapses using a Kruskal-Wallis test and found no significant difference (*X*^2^(4) = 5.093, p = .278).

**Table 9:** Comparison of MSRS-R by number of MS relapses reported on profile in the past two years

** Number of relapses in previous 2 years **	**N**	**Total MSRS-R (0–32)**
0 (none reported)	471	10.2 (5.8)
1	182	11.2 (5.9)
2	85	11.9 (6.3)
3	35	12.0 (6.7)
4 or more	43	14.5 (7.1)

### MSRS-R in retrospectively reported relapses

**Figure 3:**
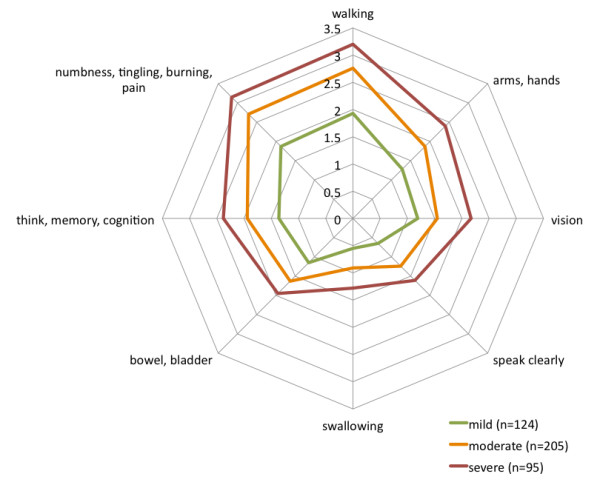
Spider plot of MSRS-R domain differences between baseline MSRS-R score and ‘most recent’ relapse, broken out by severity.

Within MSRS-R scores for retrospectively reported relapses captured actively in the survey (as opposed to passively in the patient’s site profile), Figure 3 shows the difference between MSRS-R at baseline and most recent relapse according to the perceived severity of the relapse (“mild”, “moderate”, or “severe”, N = 424). The largest differences were reported, on average, for walking, upper limb function, and the sensations (numbness, tingling, burning, and pain), followed by vision, speaking, and then swallowing.

## Discussion

The original MSRS was designed to minimize respondent burden, using a minimum number of items to cover relevant aspects of patient experience and simple, clear language in both the questions and response options. Patients have indicated in qualitative interviews that the questions are relevant to their experience, easy to understand, and easy to respond to, and provide an accurate profile of their experience of MS over time. Its deployment on PatientsLikeMe led to widespread use by thousands of MS patients, who report that using the site has produced a number of benefits including improved understanding of their condition and improved communication with their healthcare providers [5]. Following cognitive debriefing, a number of small modifications were made to produce the MSRS-R (Revised). After fielding in a survey, statistical analysis shows the MSRS-R exhibits desirable psychometric properties in terms of ceiling and floor effects, internal consistency, factor structure, test-retest, and known-group validity. Importantly, the MSRS-R correlated in expected ways with alternative measures in widespread use (MSIS-29, PDDS, NARCOMS PS, PRIMUS, and MSWS-12), suggesting acceptable concurrent validity and potential use as a research tool.

The MSRS-R has the advantage of being more concise than any of the other instruments fielded in this study; for instance, the PDDS requires a patient to read approximately 360 written words to gauge walking disability; the MSRS-R walking item has 33 words and produces very similar results. Furthermore, our analysis confirmed that the PDDS, like the EDSS it is based upon, is predominantly focused on walking; by contrast, the entire MSRS-R covers eight domains but is only 53 words long and uses a consistent response format, which makes it less burdensome for patients to read and complete.

Currently, MS trial design focuses on the frequency of relapses but is uninformed by the *nature* of these relapses, and so an attack that leaves one patient unable to walk and another unable to see are counted the same. Analysis of retrospective relapses in the current study demonstrated that the nature of relapses experienced in this population could be characterized by changes from baseline within specific domains of function using the MSRS-R. This may be useful in improving our understanding of MS characterization, progression, and response to therapy. In addition, our known group validity analysis suggested that the MSRS-R might be more sensitive to cumulative burden of disability resulting from recurrent relapses than the PDDS; further study could compare MSRS-R against other measures of cumulative burden such as magnetic resonance imaging.

Following this psychometric validation and upgrade of the existing MSRS to the newer MSRS-R, passively collected profile data in the PatientsLikeMe platform could be studied as a form of observational registry combining demographic, social networking, treatment, and symptom data. Such data would extend to a larger number of patients than described here, and to MS disease types other than RRMS, and could illuminate the real-world impact of newer therapies for MS. For instance, it may be particularly interesting to retrieve the prospective data for patients who did not report any treatment at the time of creating their account and study the amount of MSRS-R change that triggers initiation of treatment, or to gauge the effectiveness of treatment in stabilizing or reducing disability relative to similar patients who did not start treatment.

With regard to administration, although we did not explicitly test for differences between, e.g., paper-and-pencil questionnaires compared with online questionnaires, we expect that there would be no or minimal difference between data collected in these modes. The cognitive interviews did not suggest any significant difference between patients’ responses on paper and how they would have responded (or how they had responded previously) using the report tools on the PatientsLikeMe platform. The online form is two-dimensional, and no wording or format changes are required to adapt the MSRS-R, symptoms, or relapse questions for paper-and-pencil administration.

The limitations of this study are shared by many postal or internet-based questionnaire designs. We have no independent validation that respondents actually do have MS; however, as there was no incentive for participating, there would be little incentive to enter false data. Our analysis of responders found them to be a little older and more affected by MS than non-respondents; this is perhaps unsurprising given that sicker patients may be more inclined to return to PatientsLikeMe to seek support. One advantage of this data collection platform is that we can systematically describe the population of non-responders.

It is likely that the entire PatientsLikeMe population may differ systematically from the broader MS population (see [8] for a comparison with the Sonya Slifka Longitudinal MS Study), therefore these findings should be generalized cautiously. That said, our findings on the response characteristics of the comparison instruments used proved similar to their own validation studies. The response rate (19%) was relatively low but was not atypical for a survey of this online community [5]. The most significant limitation was that we lacked independent clinical assessment of disease severity from a clinician experienced in the field; we are seeking to address this in future studies.

A further limitation that underscores the difference between passively collected profile data and actively sampled survey data is the mismatch between the 345 completers (43%) who had at least one relapse recorded on their profile and the 424 completers (52%) who reported having at least one relapse when polled on the survey. Passively collected data provides a large body of longitudinal data but suffers from attrition bias; actively collected data provides a more accurate cross-section at one or a few points in time but is more costly to collect and may suffer from responder bias.

Evaluating the quality of the test-retest with a Bland-Altman plot is difficult in the absence of a gold standard and the lack of agreed standards for measurement variability in MS. It is possible that in performing the test-retest, some patients may not have clearly read the instructions to report retrospectively to the first time they completed the survey; if so, the degree of test-retest agreement reported here would be an underestimate and should be investigated further.

A copy of the MSRS-R is included as an appendix to this manuscript, and the instrument is distributed with a Creative Commons “Attribution-ShareAlike3.0 Unported” license, meaning it can be used freely (including commercially), altered, transformed, or built upon, so long as all derivative work is licensed in the same fashion and proper attribution is made (Additional file 1).

## Conclusion

The MSRS-R has been shown to be a useful tool for measuring the impact of MS and may help patients and clinicians understand the course of disease, the impact of their treatments, side effects, and relapses. It is hoped that an enhanced understanding of these aspects of MS may help improve patients' outcomes.

## Abbreviations

EDSS: Expanded Disability Status Scale; GNDS: Guy’s Neurological Disability Scale; MRI: Magnetic Resonance Imaging; MS: Multiple Sclerosis; MSIS-29: 29-item Multiple Sclerosis Impact Scale; MSRS: MS Rating Scale; MSRS-R: MS Rating Scale (Revised); MSWS-12: 12-item Multiple Sclerosis Walking Scale; NARCOMS: North American Research Committee on MS Registry; PCA: Principal Component Analysis; PDDS: Patient-Determined Disease Steps; PLM-QOL: PatientsLikeMe Quality of Life scale; PPMS: Primary Progressive Multiple Sclerosis; PRO: Patient Reported Outcome; PS: Performance Scales; PRIMUS: Patient-Reported outcome Indices for Multiple Sclerosis; QOL: Quality of Life; RRMS: Relapsing-Remitting Multiple Sclerosis; SPMS: Secondary Progressive Multiple Sclerosis; SPSS: Statistical Package for the Social Sciences.

## Competing interests

PW, TV, & MM are all current or former employees of PatientsLikeMe and hold stock / stock options in the company. The PatientsLikeMe R&D team has received research support from Abbott, Accorda, Avanir, Biogen, Genzyme, Merck, Novartis, Sanofi and UCB.

## Authors’ contributions

PW: Designed MSRS, project design, revised manuscript, statistical analysis. TV: Data analysis, reviewed manuscript. MM: Drafted manuscript, project design, cognitive debriefing, statistical analysis. All authors read and approved the final manuscript.

## Supplementary Material

Additional file 1 **Multiple Sclerosis Rating Scale (Revised): MSRS-R [ **[25]**].** Click here for file
