# Correction: The multiple sclerosis rating scale, revised (MSRS-R): development, refinement, and psychometric validation using an online community

**DOI:** 10.1186/1477-7525-11-60

**Published:** 2013-04-12

**Authors:** Paul Wicks, Timothy E Vaughan, Michael P Massagli

**Affiliations:** 1 PatientsLikeMe Inc, 155 Second Street, Cambridge, MA 02141, USA

## Correction

In our study [1], we neglected to obtain formal permission to use the correct version of PRIMUS [2]. Therefore after publication we asked the Publisher to remove the results obtained with the PRIMUS tool and the data associated with it. Although amendments were included in the pdf version of the article by the Publisher, the website xml version was left unchanged. To remove confusion, please use the corrected pdf version of the article that can be downloaded here: http://www.hqlo.com/content/pdf/1477-7525-10-70.pdf.

The authors and the Publisher apologize to the readers and the owners of PRIMUS for the inconvenience caused.
