# Supplementary material for: The multiple sclerosis rating scale, revised (MSRS-R): Development, refinement, and psychometric validation using an online community
Source: Health Qual Life Outcomes. 2012 Jun 18;10:70. doi: 10.1186/1477-7525-10-70 (PMC3502161; doi:10.1186/1477-7525-10-70)
Supplement: Additional file 1 — Multiple Sclerosis Rating Scale (Revised): MSRS-R [ [25]]. [file 1477-7525-10-70-S1.docx]

**Multiple Sclerosis Rating Scale (Revised): MSRS-R**

The MSRS-R is a quick and easy way to track your progress. Simply answer the following questions about the impact of MS on your day-to-day life. For each, select the level of disability that best represents your current condition.

|  | **No Symptoms**  No symptoms or disability in this specific area | **Some symptoms, No Disability**  Aware of symptoms but no limits on my activities | **Mild Disability**  Mild limits on my activities, but I do not need help from others or use other aids | **Moderate Disability**  Moderate limits on my activities and I sometimes need help from others or use other aids | **Severe Disability**  Severe limits on my activities and I usually need help from others or use other aids |
| --- | --- | --- | --- | --- | --- |
| **Walking** | 0 | 1 | 2 | 3 | 4 |
| **Using your arms and hands** | 0 | 1 | 2 | 3 | 4 |
| **Vision (with glasses or contacts if you use them)** | 0 | 1 | 2 | 3 | 4 |
| **Speaking Clearly** | 0 | 1 | 2 | 3 | 4 |
| **Swallowing** | 0 | 1 | 2 | 3 | 4 |
| **Bowel or bladder** | 0 | 1 | 2 | 3 | 4 |
| **Thinking, Memory, or Cognition** | 0 | 1 | 2 | 3 | 4 |
| **Numbness, Tingling, Burning Sensation or Pain** | 0 | 1 | 2 | 3 | 4 |

Citation: [25]

This work is licensed under a [Creative Commons Attribution-ShareAlike 3.0 Unported License](http://creativecommons.org/licenses/by-sa/3.0/).
